# Integrated Fault Tree and Case Analysis for Equipment Conventional Fault IETM Diagnosis

**DOI:** 10.3390/s25175231

**Published:** 2025-08-22

**Authors:** Jiaju Wu, Chuan Chen, Yongqi Ma, Ze Xiu, Zheng Cheng, Yao Pan, Shihao Song

**Affiliations:** 1 Institute of Computer Application, China Academy of Engineering Physics, Mianyang 621900, China; wujj@caep.cn (J.W.);; 2 College of Civil Aviation, Nanjing University of Aeronautics and Astronautics, Nanjing 210016, China; 3 Mobile Computing Center, University of Electronic Science and Technology of China, Chengdu 611731, China

**Keywords:** conventional fault diagnosis, digital twin, IETM, fault tree analysis, fault DM, twin data, similarity

## Abstract

Most of the failures during the actual operation of equipment are caused by improper human operation, tools, spare parts, and environmental factors. These faults are routine. Conventional faults have been validated during equipment development, testing, identification, and maintenance processes, with clear definitions and clear fault tree analysis (FTA) conclusions. Digital twins can offer rapid and interactive diagnostic capabilities for routine equipment failures. To enhance the efficiency of routine fault diagnosis and the interactive experience of the diagnosis process, this paper proposes a digital twin-based equipment routine fault diagnosis model. On this basis, considering the excellent interactivity of the Interactive Electronic Technical Manual (IETM), a conventional equipment fault diagnosis scheme based on twin data and IETM is designed. This scheme converts the equipment fault tree into an IETM fault data model (DM), which is structured and stored in a database to form a fault database. Using real-time twin data of equipment as input, the FTA method is adopted to perform step-by-step fault diagnosis and isolation guidance operation through the IETM process DM combined with fault, while providing maintenance operation guidance. When the real-time twin data of the equipment is not completely consistent with the fault information in the fault library, the case analysis method is used to calculate the similarity between the real-time twin data of the equipment and the clearly defined fault symptom information in the fault library. Based on the set similarity threshold, IETM pushes fault DMs above the threshold for corresponding fault diagnosis isolation guidance.

## 1. Introduction

With the development of high tech, information technology, and artificial intelligence technology and their widespread application in the field of equipment, high precision, automation, intelligence, and systematization are new trends in equipment development [1]. The increasingly complex structure and functions of equipment, harsh and variable operating environments, more extreme internal environments, and limited sensors have made rapid and accurate diagnosis of equipment failures a major challenge. Most of the malfunctions during the actual operation of equipment are caused by improper human operation, environmental factors, tools, and other factors. The main problem we need to solve is how to efficiently and accurately diagnose routine equipment faults using existing FTA conclusions and twin data from real-time equipment operation monitoring. Based on the fault diagnosis, we can immediately carry out fault isolation treatment, develop maintenance plans, and provide maintenance and repair guidance. IETM combines expert knowledge, virtual real interaction, multimedia demonstration, database retrieval and navigation, intelligent diagnostic analysis, and other technologies [2]. It refines and organizes knowledge of operation instructions, training procedures, testing requirements, maintenance processes, structural drawings, fault diagnosis analysis, etc., and interactively applies it to interfaces such as support systems and testing systems. It is important support equipment [3]. With the continuous development of equipment towards intelligence, automation, and complexity, the new development direction of IETM is to combine equipment status monitoring, fault diagnosis, maintenance, upkeep, and management [4]. The U.S. military standard MIL-PRF-87268/87269 [5,6] and the European standard S1000D [7,8] series standards represent the two major international standard systems of IETM. Currently, S1000D has become an international mainstream standard [9,10]. The implementation of IETM in China is mainly based on the GJB6600 standard released in 2008. This standard is a tailoring of S1000D based on the actual situation in China [10,11]. In 2003, Grieves from the University of Michigan proposed the concept of digital twins. NASA first applied digital twin technology in the Apollo program. At present, digital twin technology has been preliminarily applied in the design, maintenance, evaluation, and fault diagnosis of aerospace, shipbuilding, and other fields [12].

This article addresses the problem of rapid and accurate diagnosis of equipment routine faults with clear and defined fault modes. The concept of digital twin technology is introduced into equipment fault diagnosis, and a fault tree and case analysis integrated IETM diagnosis method for equipment routine faults is proposed. This method utilizes fault tree analysis to solve the accuracy and efficiency issues of conventional equipment fault diagnosis. The method utilizes the interactivity of IETM to enhance the user experience of the diagnostic process. The method improves the diagnostic efficiency and accuracy of similar faults by analyzing case similarity and pushing similar fault diagnosis programs.

## 2. Related Works

The equipment fault diagnosis methods include physical model-driven methods, knowledge-driven methods, data-driven methods, and hybrid intelligence methods [13,14,15,16,17,18,19]. The advantages and disadvantages of each method are shown in Table 1.

The development trend of equipment fault diagnosis is the deep integration mechanism of “physics data”, the development of diagnostic architecture with online self-evolution capability, and the establishment of industry-standardized verification benchmarks.

1. The combination of hybrid intelligence, digital twins, and deep learning has become a new hot topic.

2. Explanatory driving technological innovation: SHAP [20], LIME [21], Grad-CAM [22], and other explanatory technologies are accelerating their integration into diagnostic systems.

3. Popularization of edge cloud collaborative architecture: Lightweight models (such as TinyML with <1 MB parameters) are deployed on edge nodes to achieve millisecond-level real-time diagnosis; the cloud is responsible for model retraining and knowledge accumulation.

4. Small sample learning breaks through the data dilemma: Federated learning and transfer learning significantly alleviate the problem of data scarcity, and the technology of synthesizing fault data using Generative Adversarial Networks (GANs) is being applied in equipment fault diagnosis.

The conventional faults of the equipment studied in this article have known fault modes and clear diagnostic knowledge. Therefore, based on the comparison of the advantages and disadvantages in Table 1, choosing the fault tree analysis method is the most suitable. Combining the interactivity of IETM and real-time sensor data for real-time fault diagnosis reasoning can ensure the accuracy of equipment fault diagnosis while improving user experience and real-time performance, facilitating immediate maintenance measures.

## 3. Equipment Fault Diagnosis Based on Digital Twins

**Figure 1:**
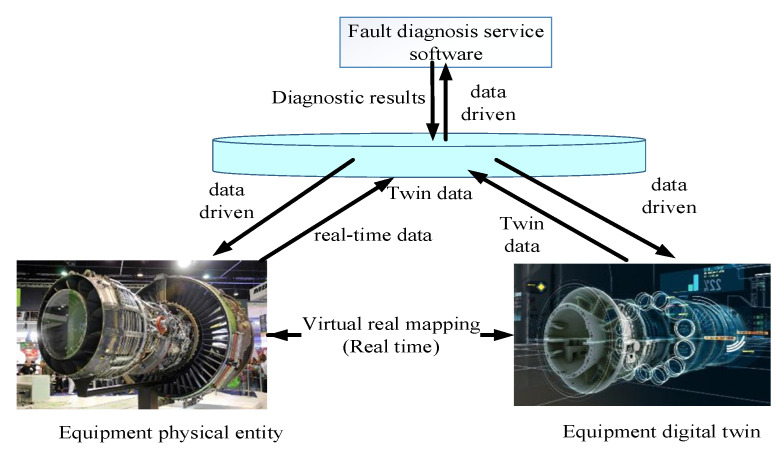
Equipment fault diagnosis model based on digital twins.

The equipment fault diagnosis model based on digital twins is shown in Figure 1, which includes physical entities of equipment, digital twins of equipment, twin data, and fault diagnosis service APP software, such as Oil Cloud V3.0, NSK Doctor V1.0, AutoFTA V4.0, Industrial Control Master v1.0.17, etc.

The physical entity of the equipment consists of real physical equipment, monitoring/detection devices (such as sensors), and corresponding installation and operation environments. An equipment digital twin is a real-time mapping virtual digital model of physical equipment in the digital space. Twin data includes historical factory data, historical data, performance data, monitoring/testing data, real-time monitoring/testing data, and virtual digital model data of equipment physical entities [9]. Fault diagnosis service software is based on twin data, such as equipment historical data, real-time information, model data, and FTA data, as well as expert knowledge, rule judgment, fault analysis, machine learning, and other fault diagnosis methods. Software is provided for use as a service. The diagnostic service software takes real-time twin data of equipment as input, uses some fault diagnosis methods for calculation, finds the corresponding relationship between equipment degradation laws and equipment parameter data and characteristics, and determines the real-time status of equipment. The status data will be fed back in real time to the digital twin of the virtual space. The equipment digital twin needs to analyze the twin data and its technical status, update the relevant data of the virtual digital model, and display the real-time status of the equipment in a high-fidelity manner. When the equipment maintains good technical condition, real-time twin data is collected and stored in the twin data center. Fault diagnosis services determine the normal operating status of the equipment based on relatively simple knowledge-based rules and provide feedback. When the equipment monitoring/testing data contains certain fault symptoms, the operational data, environmental data, and human operation data will be automatically saved to the twin data center. The fault diagnosis service software calls on expert knowledge, machine learning algorithms, and intelligent diagnostic models for predictive diagnostic calculations. If there is an error between the calculated results and the actual situation, the parameters and calculation methods of the diagnostic model can be adjusted and recalculated until the required diagnostic accuracy and precision are achieved.

**Figure 2:**
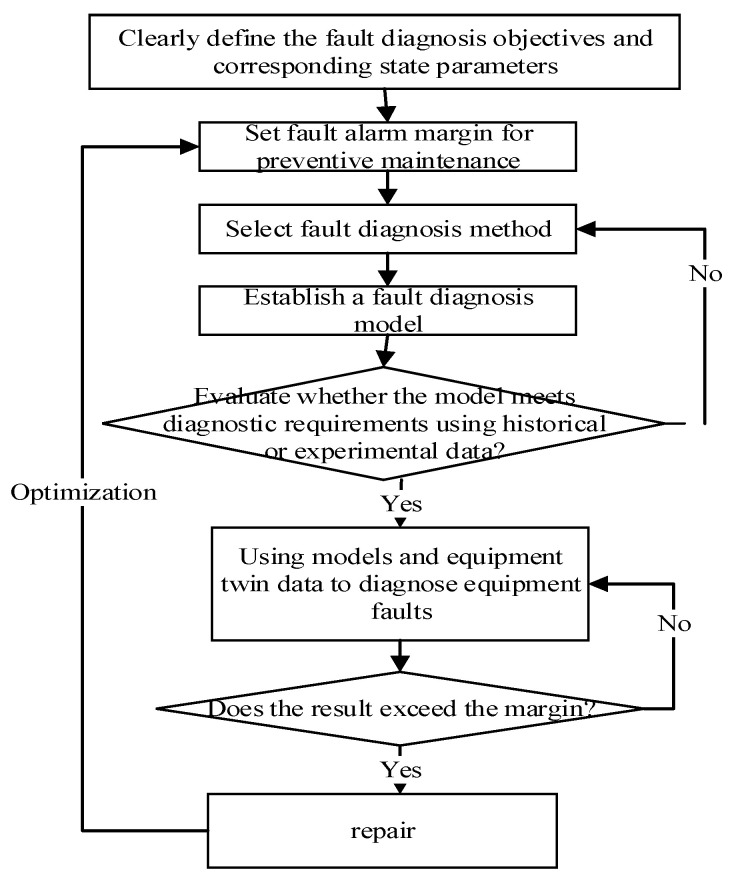
Equipment fault diagnosis and prediction process based on twin data.

The modeling and optimization process of equipment fault diagnosis prediction based on twin data is shown in Figure 2. Based on the equipment diagnostic model, twin data is used for calculation and prediction, thereby inferring the health status of the modified equipment in a certain period in the future and detecting potential faults in real time. Based on the fault prediction results, we can take maintenance measures in advance to avoid accidents caused by faults.

The specific steps are as follows:

Firstly, based on the composition, characteristics, and operational status of the equipment system, the fault diagnosis objectives are defined, such as equipment systems, subsystems, or critical components.

Next, based on the characteristics of the diagnostic target, parameters that significantly affect the operational performance and functionality of the equipment are set as diagnostic parameters.

Then, according to the maintenance strategy, we will set a fault alarm margin for preventive maintenance.

Next, fault diagnosis and prediction methods will be selected, such as statistical analysis methods, rule-based reasoning methods, information fusion methods, case-based reasoning methods, and machine learning methods.

Furthermore, we have established a predictive model for equipment fault diagnosis.

Next, historical or experimental data will be used to evaluate whether the diagnostic prediction model can achieve performance, including evaluation indicators such as prediction accuracy, generalization degree, and robustness. If the diagnostic model fails to achieve its effectiveness, we will continue to optimize the model. If the diagnostic model can achieve the desired effect, we will deploy it to the digital twin system of physical equipment.

Finally, real-time twin data of equipment (performance monitoring data, detection data, etc.) will be used for equipment fault diagnosis calculation and prediction. When the calculation result is within the set fault alarm margin range, the equipment is in normal operation. The monitoring and detection data at later times will continue to be collected and used for equipment fault diagnosis and prediction. When the calculation result exceeds the set alarm margin, we will take corresponding preventive maintenance measures and optimize the fault alarm margin based on the maintenance situation (such as maintenance time, maintenance cost, post-repair quality, etc.).

## 4. Equipment Conventional Fault Diagnosis Based on Twin Data and IETM

### 4.1. IETM Diagnostic Method for Equipment Conventional Faults

**Figure 3:**
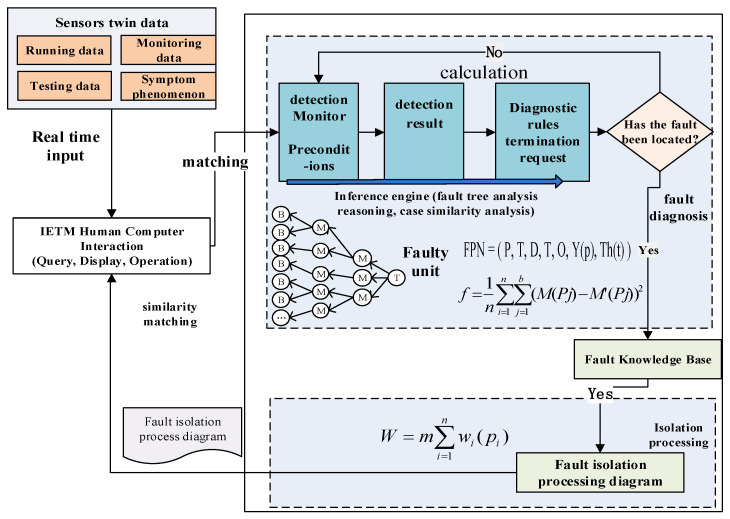
Integrated fault tree and case analysis for equipment conventional fault IETM diagnosis.

The fault tree and case analysis integrated IETM diagnosis method for equipment conventional faults is shown in Figure 3. Firstly, the fault tree of routine faults that are clearly defined during the equipment development, testing, and identification process is converted into the fault data module of IETM according to the mapping rules. These fault data modules are structured and stored in a database to form an IETM fault library. Next, the fault tree inference analysis process is transformed into the IETM process data module. Then, real-time twin data is used as diagnostic input, and the IETM process data module organizes the execution of the diagnostic inference process. During the execution of diagnostic reasoning, specific fault data modules corresponding to intermediate events and basic events are called to realize the diagnosis and isolation guidance of equipment conventional faults. When a completely matching fault data module cannot be found in the IETM fault library, calculate the similarity between real-time twin data and fault library cases, and recommend similar fault diagnosis program data modules greater than the set threshold to carry out equipment fault diagnosis.

The real-time twin data collected by equipment sensors is the input for fault diagnosis. Sensors include four categories: contact sensors, non-contact sensors, wireless sensors, embedded sensors, and intelligent components. Their data collection methods are as follows. The data acquisition process of contact sensors such as strain gauges, thermocouples, piezoelectric sensors, and speed sensors is as follows: sensor → signal conditioner (amplifier/filter) → wired transmission → PLC/data acquisition card → digital twin platform. Laser radar, infrared thermal imager, industrial camera, etc., perceive targets remotely through electromagnetic waves, light, or sound waves. The data acquisition process of non-contact sensors without physical contact is sensor → edge computing node (preprocessing point cloud/image) → 5G/fiber → cloud digital twin. Wireless sensors such as RF chips, vibration/temperature/humidity sensors, microprocessors, etc., use magnetic nodes to absorb the device surface, are powered by batteries, and sensor data is uploaded to the cloud platform through 4G/5G. Embedded sensors and intelligent components are directly connected to the digital twin platform through industrial protocols such as OPC UA and Modbus TCP.

The process DM of IETM can realize the sequential definition of the interactive diagnosis process for complex equipment faults and control the flow of diagnostic programs. The process DM can implement fault diagnosis reasoning logic based on real-time data through external testing program interfaces. The fault DM can define equipment faults and form a fault list. The fault DM provides a structured definition, retrieval, rule judgment, and interactive presentation of fault tree knowledge, forming a fault knowledge base.

The fault isolation program implements interactive fault isolation guidance operations. For routine faults with clear causes, propagation modes, and phenomena, the fault isolation and maintenance plan will be validated by the equipment development party through testing and will form a fault manual for delivery along with the installation, such as the Aircraft Fault Isolation Manual (FIM), Aircraft Maintenance Manual (AMM), etc. The elements and attribute definitions of the IETM fault DM can structure the fault products, fault codes, fault phenomena, fault modes, fault causes, occurrence conditions, operation processes, diagnostic methods, diagnostic processes, etc., of conventional equipment faults into a fault library. The inference rules formed by the forward and backward inference mechanisms can be defined as interactive guidance operation sequences and decision rules in the IETM process DM. Combining historical fault data of similar equipment or equipment, the process DM and fault DM can achieve interactive fault diagnosis in the object domain and equipment twin data.

The specific events of the equipment fault tree can be transformed into a fault DM, forming a fault knowledge base, including the phenomena of faults, judgment rules, possible causes, equipment information, handling measures, and isolation operation procedures [23]. The process DM contains an interactive process structure, which is equivalent to the flow of events in each node of the fault tree. It is used to describe the complete process of fault isolation and diagnosis, and specifies the sequential relationship between different DMs based on variables and different step information of the same DM. The process DM is used to organize the specific implementation process of fault diagnosis and isolation procedures. The twin data of the equipment is used as input for diagnosis. By matching and comparing twin data with the equipment fault knowledge base, IETM can find the fault diagnosis program corresponding to the fault mode or similar cases, and perform fault diagnosis. Based on the diagnostic results, IETM calls the corresponding fault isolation boot program to implement the fault isolation operation of the equipment. Finally, IETM provides diagnostic results or isolation operation conclusions to equipment users through a human–computer interaction interface, and stores successful fault diagnosis cases in the IETM case library for similar case queries.

### 4.2. Fault Tree Analysis

**Figure 4:**
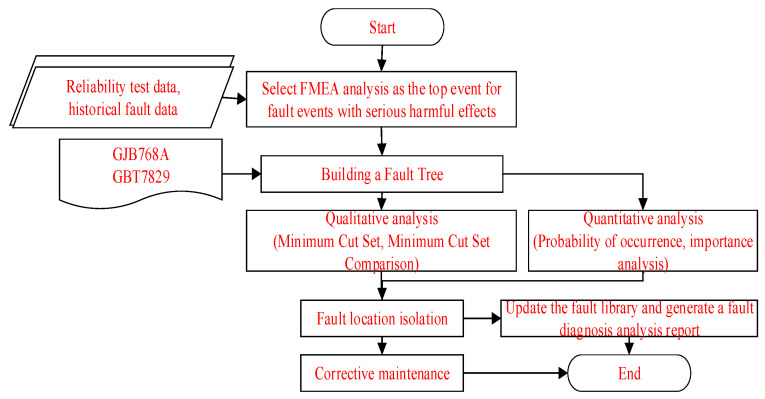
Fault tree structure diagram.

A fault tree is a logical diagram displaying the causes of failure in an inverted tree structure [24], describing the logical relationship between equipment failures and the causes of failure in hardware, software, materials, processes, operating methods, personnel, and other factors. FTA is an analysis method that starts from the root node (top event) of a tree to intermediate nodes (intermediate events) and leaf nodes (basic events), until all leaf nodes that cause the root node event are found. The specific method of FTA is to subdivide equipment faults into sub-faults that may cause faults, and then decompose the sub-faults into smaller sub-faults, decomposing and delineating the scope layer by layer, from top to bottom, and refining step by step [25,26]. We analyze the specific causes and relationships of faults in each component and draw a fault tree through software. The software connects various faults based on the connections between faults and between faults and the system, and constructs a fault tree model, as shown in Figure 4. When a fault occurs in the equipment system, the fault tree model serves as a behavior prediction and inference model. Based on the fault tree model, the fault is identified step by step to find the cause of the equipment system fault, locate the fault logic replaceable unit (LRU), and respond in real time to the fault isolation processing program.

**Figure 5:**
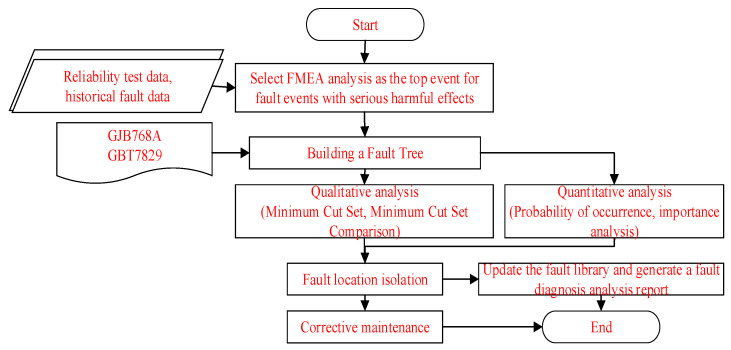
Fault tree analysis process.

FTA generally uses a combination of qualitative and quantitative methods, and the analysis process is shown in Figure 5.

**Figure 6:**
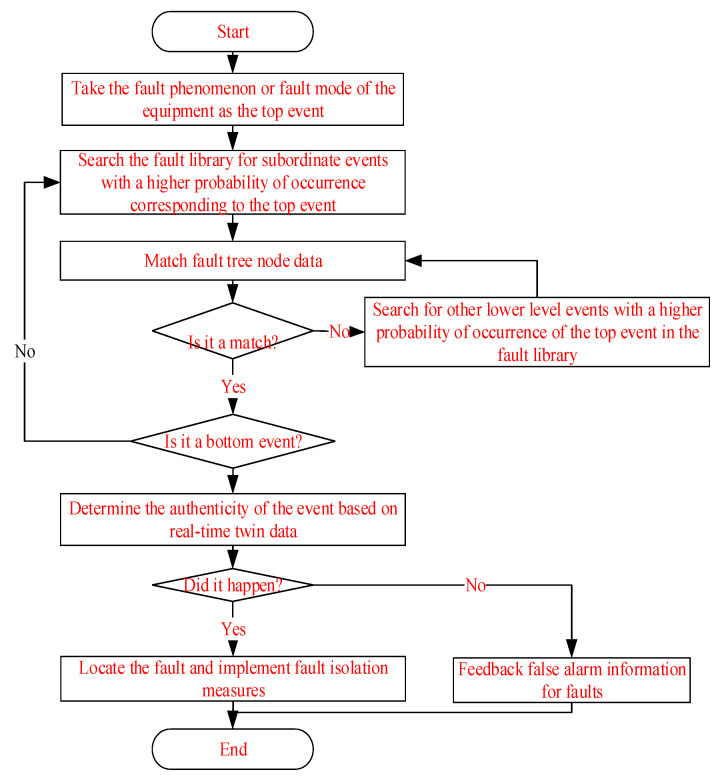
The diagnostic reasoning process of the fault tree.

Qualitative analysis can obtain the basic event set that triggers the top event, namely the minimum cut set. The correlation matrix, up and down methods, and Boolean operations can all solve for the minimum cut set. Quantitative analysis can calculate the probability of the top event occurring in the fault tree model, locate the location of the fault, take corresponding fault isolation measures, and update the fault case library. The diagnostic reasoning process is shown in Figure 6.

The reasoning strategy in the diagnostic process is opposite to fault tree analysis, which infers the cause of the occurrence based on the phenomenon, and achieves localization and isolation.

The basic event state includes two types: fault and normal, as shown in Equation (1).

(1) $$B_{i}=\left\{\begin{array}{c} 1,\;\;\;\;\mathrm{Fault}\;\mathrm{state}\\ 0,\;\;\;\;\mathrm{Normal}\;\mathrm{state} \end{array}\right.$$

Assuming the probability of the basic event Bi is PBi, the prior probability of the basic event is obtained from historical operating data or experimental data statistics. According to the conditional probability formula, the probability of Mi corresponding to OR gates B_1_, B_2_, …, B_i_ is shown in Equation (2).

(2) $$P_{Mi}=1-\prod_{1}^{i}\left(1-P_{Bi}\right)$$

The probability of Mi corresponding to gates B_1_, B_2_,…, B_i_ is shown in Equation (3).

(3) $$P_{Mi}\;=\prod_{1}^{i}P_{Bi}\;$$

The probability of Mi corresponding to XOR gates B_1_, B_2_,…, B_i_ is shown in Equation (4).

(4) $$P_{Mi}=\prod_{1}^{i}\left({1-P}_{Bi}\right)\;$$

The probability of M_i_ corresponding to non-gate B_i_ is shown in Equation (5).

(5) $$P_{Mi}=1-P_{Bi}\;$$

The probability ratio of B_i_ to the top event is shown in Equation (6).

(6) $$P_{Bi/T}=\frac{P_{Bi}}{P_{T}}\;$$

The probability ratio of Mi to the top event is shown in Equation (7).

(7) $$P_{Mi/T}=\frac{P_{Mi}}{P_{T}}$$

The magnitude of probability proportion reflects the degree of impact of an event. To achieve rapid diagnosis and localization of faults, the inference sequence of the fault diagnosis sub-process is designed based on the probability proportion of intermediate events to top events. The reasoning process is executed first for significant intermediate events, and then for less important ones. The diagnostic sequence of basic events is also determined by the order of probability proportion.

### 4.3. Case Similarity Analysis

Partial fault phenomena, the fault database does not have accurately matched fault modes and isolation procedures. In response to this situation, we generally use the case similarity calculation method to calculate the similarity between the equipment fault phenomenon, operating data, environment, and experience cases in the fault library. Then, based on the similarity threshold, the IETM viewer software V1.0 matches the fault diagnosis reports and isolation programs of experience cases in the knowledge base that exceed the threshold, to achieve the diagnosis and isolation of routine equipment faults.

Similarity calculation generally uses the nearest neighbor algorithm, KNN algorithm, or neural network classification algorithm for matching similar fault phenomena [27,28]. Firstly, calculate the similarity between the experience cases in the fault library and the current fault phenomenon. Then, set a similarity threshold (e.g., 90%) to calculate the similarity between the occurrence conditions and the environment. If the similarity is greater than or equal to the threshold, it is recommended to isolate and diagnose the fault DM of the case. Otherwise, it is not recommended. When the diagnosis results are consistent with expectations, the diagnosis is completed. When the diagnosis result is inconsistent with the expected result, manual diagnosis should be carried out. Finally, the diagnostic process is edited into a fault DM and added to the fault library, updating the case library.

This article uses KNN for similarity calculation, and the similarity calculation formula of the KNN algorithm [29,30] is shown in Equation (8).

(8) $$S\left(T,\;S\right)=Sim\left(\sum_{i=1}^{n}f\left(T_{i},\;S_{i}\right)w_{i}\right)$$

In the equation, T represents the current fault case. S indicates that there are already historical cases. N is the number of parameters or text segmentation used to describe the fault case. I represents the i-th parameter or text segmentation. $f\left(T_{i},\;S_{i}\right)$ is the similarity function between the target case and the historical case in the i-th parameter or text segmentation. Generally, text segmentation technology is used first for segmentation, and then Euclidean distance calculation is used. Wi represents the weight of the i-th attribute.

### 4.4. Equipment Fault Diagnosis Based on IETM

**Figure 7:**
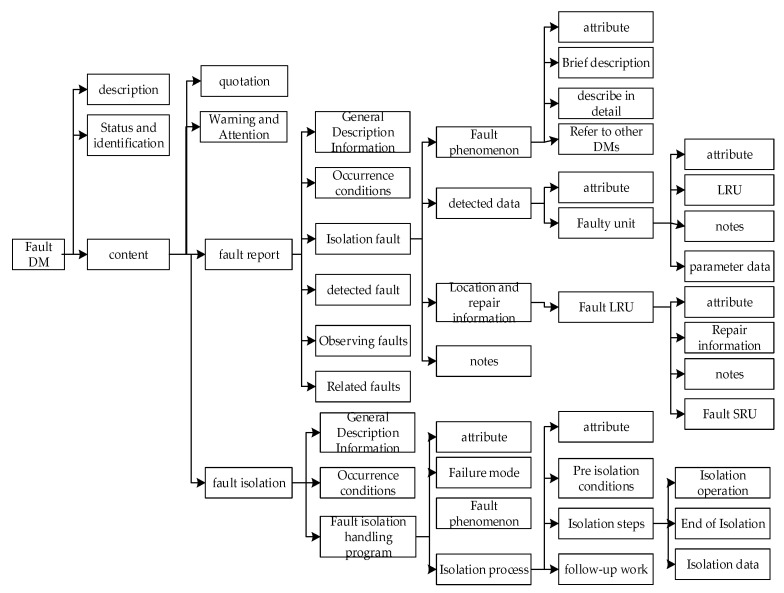
Data structure of fault DM.

In IETM, both S1000D and GJB6600 standards define fault DMs and process DMs. Combining these two types of DMs can achieve interactive fault diagnosis and isolation guidance operations for conventional equipment faults [31]. The process DM mainly implements interactive and sequential control, similar to fault tree analysis. The fault DM includes fault reports and isolation programs, and its DM is shown in Figure 7.

Fault report is equivalent to a list of equipment faults, including fault name, fault description (function, performance, quality, control, online status, circuit switch status, etc.), general information (components, fault diagram, fault video multimedia, warning, attention, annotation, data table, data grouping, hotspots, etc.), possible causes, occurrence conditions, working time, occurrence time, environmental conditions, onboard monitoring indicators, discoverers, maintenance program index, etc. From the perspective of discovery and diagnosis methods, faults are classified into four categories: isolation, detection, observation, and related faults for a detailed definition. Isolation fault refers to a fault that is simple, can be directly located, immediately isolated or repaired, and isolated based on real-time detection data, monitoring data, and judgment rules. It can be directly repaired by calling the maintenance program. Fault detection refers to the process of identifying multiple causes of a fault, which requires the use of fault isolation or detection programs for each possible factor. Based on real-time detection data, monitoring data, and judgment rules, fault analysis is conducted to determine the location of the faulty component and initiate corresponding maintenance procedures for repair work. Observation of faults refers to faults discovered by equipment maintenance personnel, such as corrosion, bulging, rusting, deformation, dents, etc. Associated failure refers to a component failure that may cause cascading failures in other components or subsystems. The isolation program includes general descriptive information (component, fault diagram, fault video multimedia, warning, attention, annotation, data table, data grouping, hotspot, etc.), preconditions (product master data (interval threshold, storage area, data access control, work area, task duration, etc.), operating environment, personnel, technology, support equipment, supply, spare parts, safety, etc.), isolation main program (operation steps, operation process, operation attention warning, isolation activities, detection data (diagram, multimedia, data table), isolation judgment results, maintenance program reference, replacement program reference, etc.), end program, etc.

### 4.5. Mapping Fault Tree and Diagnostic Reasoning Process to IETM DM

According to the composition of the fault tree, taking the cause of the fault symptoms as the condition and the fault symptoms as the conclusion, the fault diagnosis process is transformed into the inference rules of “IF A THEN B”. The rule-based fault tree jump traversal process can be transformed into inference logic and described using XML language. The rule of “IF condition THEN conclusion” can be placed in the DM of IETM [32]. The correspondence between the fault tree model, XML language, and IETM DM elements is shown in Table 2.

During the conversion process, the fault tree content corresponds to the fault DM in IETM, and the complex fault tree analysis process corresponds to the <dmRef> and <dmNode> elements of the process DM. The door relationship corresponds to the <dmIf>, <dmThenSeq>, and <dmElseSeq> elements of the process DM. The fault tree inference process corresponds to the <dmSeq> of the process DM, which is used to determine the sequence of steps, dialog interactions, external program requests, and conditions. The conditions and parameters of inference correspond to the <variableDeclarations> element record of the process DM. Human–computer interaction is implemented using the <dialog> element. There are various ways of human–computer interaction between humans and logic engines, such as menus, buttons, message boxes, and dialog boxes. The <externalApplication> element is used to introduce external detection or monitoring programs, converting the fault tree analysis process into the fault diagnosis process and fault DM of IETM, achieving interactive fault diagnosis [33].

### 4.6. Implementation of IETM for Equipment Conventional Fault Diagnosis Based on Twin Data from Sensors

**Figure 8:**
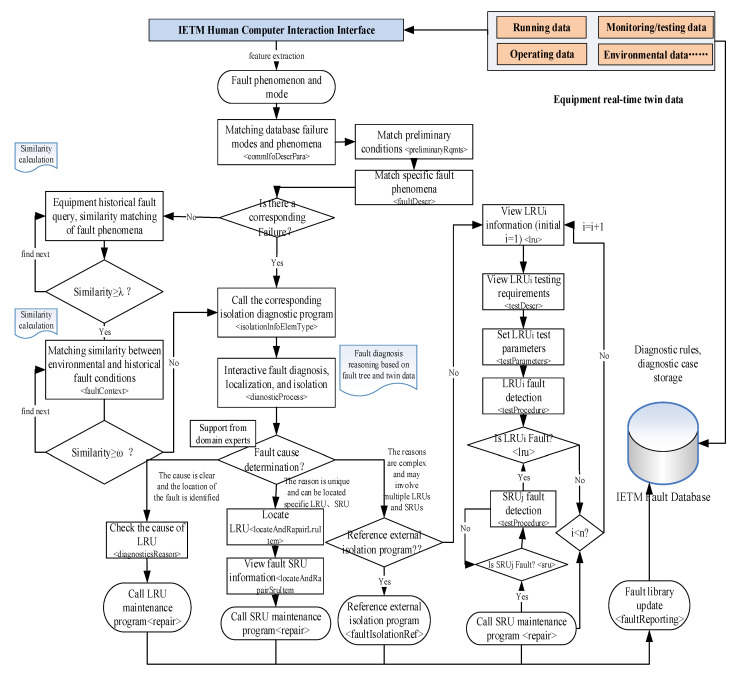
The implementation process of equipment conventional fault IETM integrating fault tree and case analysis.

The implementation of IETM fault diagnosis by integrating fault tree and case analysis is shown in Figure 8. Equipment real-time twin data (real-time detection data from sensors, monitoring data, operational data, environmental data, etc.) is used as input for routine fault diagnosis. The fault tree of equipment routine faults is made into a fault DM and stored in the IETM fault knowledge base. Using real-time twin data as input, enter the IETM fault diagnosis software system through the IETM human–computer interaction interface.

The IETM software has a built-in feature extraction method that extracts fault phenomena or features from real-time twin data. The software simultaneously queries whether there are fault reports in the fault library that contain the fault characteristics or symptoms. If the query is unsuccessful, the equipment is in normal condition and has good performance. The <faultDescr> attribute description in <faultReporting> element can identify the same or similar fault phenomena or symptoms. So this fault symptom or fault phenomenon data serves as the root node of the fault tree, which is the top event. Then, based on the fault mode or fault code corresponding to the top event, find the corresponding fault diagnosis isolation program <faultIsolation> in the fault library. Calculate the similarity between fault phenomena, fault knowledge, and historical cases through the fault library knowledge or case recognition algorithm built into IETM. Determine the importance of fault tree nodes and determine the priority of each event in the fault tree based on the similarity value. Finally, forward reasoning is performed according to priority to obtain the corresponding leaf event (bottom event) of the fault occurrence, achieving the troubleshooting and diagnosis of equipment routine faults [34].

When the real-time twin data of the equipment contains some fault symptoms, the IETM software queries its own fault library and matches the faults. Using the description of fault symptoms as input to IETM, IETM extracts keywords or fault codes of fault symptoms and searches for fault cases in the fault library. The fault description of the <commIfoDescrPara> attribute under the <commInfo> element general description for software query fault cases is a phenomenon detected by equipment monitoring/detection or input content on the human–computer interaction interface. If there is a complete match, the IETM software will continue to check whether the preliminary requirements for the occurrence of the fault are consistent through the <preliminaryRqmts> element, including environmental, operational, regional, and other information. The IETM software will confirm that the real-time fault symptoms are consistent with the cases in the fault library. If they are consistent, the software compares the specific descriptions of the four types of faults in order to find the specific fault type. Based on the specific type of fault phenomenon, symptoms, occurrence conditions, etc., the corresponding <diagnosticProcess> fault diagnosis process is called for fault diagnosis isolation. Observable faults can be isolated in real time based on observation. Other faults can be diagnosed by describing the faulty part information based on <detectionInfo> and diagnosing the isolation information based on <isolationInfo>. The diagnostic isolation process <isolateDetectedFault> provided in the fault isolation information implements fault diagnosis isolation based on detection/monitoring data. If there is inconsistency, calculate the similarity between the fault phenomena, occurrence conditions, and historical cases contained in the twin data. When the similarity is greater than or equal to the set threshold λ (e.g., 90% or 85%), calculate the occurrence condition similarity. When the similarity of the conditions is greater than or equal to the set condition ω (such as 90% or 85%), it is considered that the current equipment failure is consistent with the cases in the fault library. The IETM software automatically pushes the diagnostic isolation processing program <isolationInfoElemType> for cases in the fault case library to implement fault diagnosis isolation work.

The detailed process of fault diagnosis is described using <diagnosticProcess>. Firstly, the IETM software calculates and compares the input twin data with the built-in fault diagnosis rules. Domain experts can also be introduced to determine observation faults. When the fault is clear, the LRU fault location is determined, and the repair is simple: directly check the cause of the LRU fault, and then call the LRU unit repair program. If it can be directly determined that the equipment system failure is caused by a single SRU or LRU, and no further detection and monitoring data is needed to assist in locating the fault, the faulty LRU unit can be directly located through <locateAndRepair>. Further fault SRU (shop replaceable unit) unit is located through <locateAndRepairSruIetm>. The <repair> maintenance procedure for SRU units or LRU units will be used to carry out SRU unit maintenance. When the factors causing the malfunction are complex, there may be multiple LRU faulty units or multiple SRU faulty units. Generally, the <faultIsolationTest> testing program is executed to further diagnose and detect faults using the <isolateDetectedFault> program. Firstly, apply the <faultIsolationTest> procedure to perform fault diagnosis and detection on the LRU unit. Based on the detection data, locate the faulty LRU unit. Apply the <faultIsolationTest> procedure to perform fault diagnosis and detection on all SRU units of the LRU unit, and locate the specific faulty SRU unit. Call the corresponding repair program of the SRU faulty unit for maintenance. The <faultIsolationTest> procedure test program defines the test name, test requirements, and external test programs that can be referenced through <testDescr>, and sets the test parameters, testing conditions, testing equipment, and other information for LRU and SRU units through <testParameters>. The <testProcedure> program implements the execution of testing programs, conducting detailed inspections of possible faulty components, and determining whether they are faulty components based on inspection data and judgment rules [35]. After successfully completing equipment fault diagnosis and isolation positioning, update the fault phenomena, environmental data, etc., in the twin data to the fault cases in the fault library for recommended use in similar cases in the future. After completing the fault diagnosis and location, the isolation operation of the faulty component is carried out using the Fault Isolation Procedure. The isolation operation process is generally guided through the <action> under <isolationStep>. The human–machine interaction during the isolation operation is achieved through Q&A <yesNoAnswer> and selection <listOfChoice>.

The conventional fault diagnosis of equipment based on twin data is built into the IETM reader through software. The fault diagnosis reasoning process that integrates fault tree and case analysis is built into the IETM software. To improve the diagnostic effect during interaction, the software is implemented in Java language, and the fault diagnosis process diagram and fault tree are drawn through the mxGraph plugin. The virtual digital model display is implemented using WebGL technology [6]. The results of fault diagnosis can be promptly fed back to the virtual digital twin model of the equipment, where the fault situation of the equipment is displayed and fault alarm reminders are sent. At the same time, the repair program stored in IETM is called to guide the maintenance work.

## 5. Experiment

### 5.1. Fault Case

**Figure 9:**
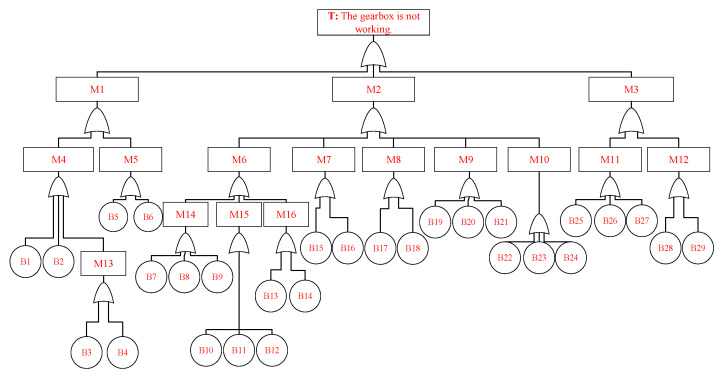
The aviation gearbox fault tree.

This article uses the fault diagnosis process of a certain aviation gearbox as an experimental case, and the fault tree is defined as shown in Figure 9 and Table 3.

The probability of occurrence of basic event faults in the gearbox is shown in Table 4. The top event of the fault tree is that the gearbox is not working. The first-level intermediate events include clutch slippage, component failure, and oil leakage. Secondary intermediate events include friction plate failure, oil hole blockage, gear failure, bearing failure, shaft failure, box rupture, fastener failure, seal failure, and joint surface failure. The third-level intermediate events include high temperature, gear tooth fracture, tooth surface wear, and bonding. The basic events include 29 categories, such as poor friction plate material, friction plate wear, fatigue fracture, overload fracture, etc. [36].

The real-time data of the gearbox collected at time t is shown in Table 5.

### 5.2. Diagnosis Process

From Figure 9, it can be seen that there are 29 types of minimum cut sets in the fault tree of the gearbox that is not working. The probability of the top event occurring is calculated as 0.0550 using Formula (2), and the probability ratio of the basic event to the top event is calculated using Formula (6), as shown in Table 6.

The probability of the occurrence of intermediate events and the probability proportion of the top event calculated by Formula (7) are shown in Table 7.

The fault diagnosis isolation program for basic events is converted to the IETM fault DM. The fault diagnosis inference process is converted to the IETM process DM. The list of IETM DMs is shown in Table 8 and Table 9. The order of fault diagnosis reasoning is sorted from high to low according to the probability proportion of the top event to the event.

The diagnostic reasoning process for the malfunction of the gearbox is executed in the following order:

$$\begin{array}{c} T\to M_{2}\to M_{6}\to M_{16}\to B_{14}\to B_{13}\to M_{15}\to B_{11}\to B_{10}\to B_{12}\\ \;\;\to M_{14}\to B_{9}\to B_{8}\to B_{7}\to M_{1}\to M_{5}\to B_{11}\to B_{12}\to M_{4}\to B_{2}\\ \;\;\to B_{1}\to M_{13}\to B_{3}\to B_{4}\to M_{3}\to M_{11}\to B_{26}\to B_{27}\to B_{25}\\ \;\;\to M_{2}\to M_{9}\to B_{20}\to B_{21}\to B_{19}\to M_{7}\to B_{15}\to B_{16}\to M_{8}\\ \;\;\to B_{17}\to B_{18}\to M_{10}\to B_{23}\to B_{22}\to B_{24}. \end{array}$$

### 5.3. Result Analysis

The real-time twin data collected by the gearbox at time t is used as input, and the fault diagnosis results obtained by integrating fault tree analysis and case similarity calculation are shown in Table 10.

IETM calls the fault DM in sequence through the process DM for responsive interactive fault diagnosis, identifies the cause of the fault, and locates the component where the fault occurred. Finally, IETM calls the fault repair program to guide the maintenance work.

This experiment uses an Intel I7 processor, with a traversal query and rule matching time of 0.256 s. From the fault diagnosis results in Table 10, it can be seen that the accuracy of equipment conventional fault diagnosis using the IETM integrated fault tree analysis is 100%. Therefore, the method proposed in this paper is suitable for online real-time fault diagnosis of conventional faults at the component and subsystem levels. The determination of fault modes and the clear definition of fault trees in IETM conventional fault diagnosis is actually a process of traversal, querying, and rule comparison. As long as the fault tree is defined clearly enough, the diagnostic rules are detailed enough, and the types and quantities of twin data collected are sufficient, and the traversal, rule matching, and comparison are strictly carried out in the order of probability proportion, the accuracy of fault diagnosis can theoretically reach 1. When the real-time twin data input does not match the definition of the fault library, the similarity between the fault phenomenon and the historical fault symptoms in the fault library is calculated. For example, if the input “tooth surface corrosion” is not completely consistent with the fault mode in the fault library, and the fault library has “B_12_ tooth surface corrosion and wear”, and the similarity with the input of “tooth surface corrosion and wear” is higher than the threshold of 80%, the B_12_ tooth surface corrosion and wear DM is automatically recommended for fault diagnosis.

When the complexity of equipment is high, the fault factors are complex, and there will be many nodes in the fault tree for diagnosis and inference traversal. The inference rules are complex, the traversal process is lengthy, and the fault diagnosis time will be relatively long. Therefore, it is necessary to improve hardware performance or adopt intelligent fault diagnosis methods to achieve real-time online fault diagnosis of complex equipment. When the complexity of the equipment system is high, the fault diagnosis program can be deployed on high-performance machines (such as adding high-performance computing cards, computer clusters, distributed computing, etc.) to solve the problem of computational time overhead.

The fusion of fault data and case analysis for equipment routine fault diagnosis mainly achieves real-time diagnosis of equipment routine faults. Equipment routine failures are the eyes of Equipment Prediction and Health Management (PHM) to perceive the “present” and the foundation of PHM. The results of fault diagnosis provide an indispensable data foundation and verification basis for predicting the “future” and making management decisions. The real-time diagnosis method for routine equipment faults can be combined with equipment health status prediction algorithms to achieve a transition from passive fault maintenance to active health management.

When the real-time twin data collected by sensors is not perfect, in order to ensure the reliability and robustness of the fault diagnosis method, the optimization of the fault diagnosis method includes four aspects: data preprocessing, adopting an adaptive inference mechanism, multi-source evidence fusion decision-making to form diagnostic conclusions, and real-time feedback optimization on the line. In terms of data preprocessing, two main methods are used: noise suppression and missing data compensation. Usually, sliding window filtering (such as the Savitzky–Golay filter, which smooths high-frequency noise and preserves fault feature trends), non-stationary signal wavelet threshold denoising (separates noise components, such as Daubechies wavelet), and outlier removal based on Isolation Forest or the 3 σ principle are used for noise suppression. The methods for compensating missing data include using historical data of similar equipment to construct a regression model (such as random forest regression) to predict missing values, integrating redundant sensor data through Kalman filtering or D-S evidence theory to reduce the impact of single point failures, and using GAN or VAE to generate synthetic data that is consistent with equipment operating conditions. In terms of diagnostic reasoning, the fault tree is flexibly extended by converting its Boolean logic into probability calculation, and introducing temporal logic gates (PAND, SEQ) to tolerate data delay/disorder. A case similarity matching method with tolerance is introduced to improve the reliability and robustness of diagnosis. In terms of multi-source evidence fusion decision-making, we generally use fault trees, Bayesian networks, and D-S fusion to obtain the final confidence level and provide diagnostic reliability. Finally, in terms of feedback online optimization, digital twin systems generally compare diagnostic results with subsequent maintenance records and automatically correct fault tree probability parameters/CBR case libraries. New noise patterns, such as periodic noise caused by electromagnetic interference, are also stored in the feature library to enhance future recognition capabilities.

### 5.4. Engineering Application

**Figure 10:**
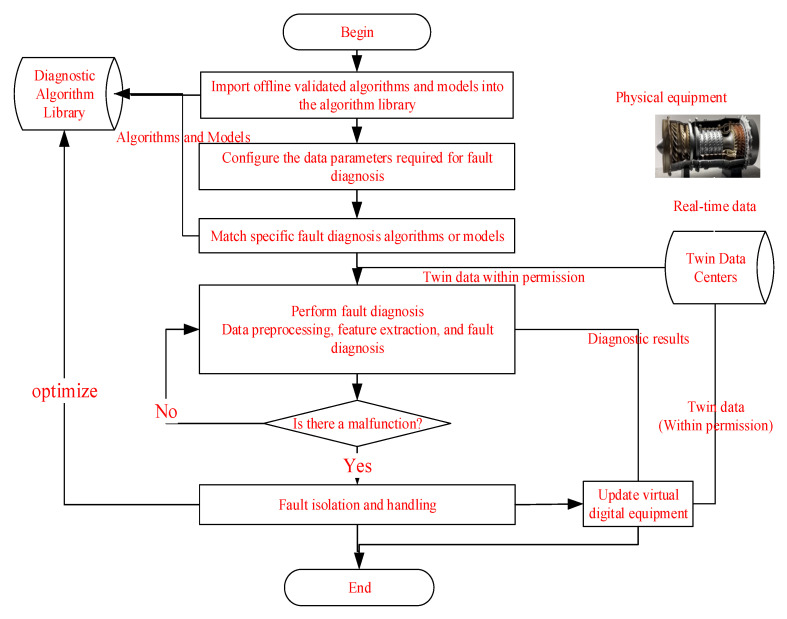
Implementation process of twin data-driven fault diagnosis method in engineering applications.

The engineering implementation application of real-time twin data-driven fault diagnosis based on sensors is shown in Figure 10. Firstly, the fault diagnosis algorithms for various fault modes or combination modes verified by historical data will be imported into the maintenance support digital twin platform to form a fault diagnosis algorithm library. Secondly, based on the characteristics of the equipment and the twin data, configure the fault diagnosis parameters to be calculated for the equipment, subsystems, or components. Then, based on the characteristics of the equipment, subsystem, or component, match the corresponding fault diagnosis algorithm from the diagnostic algorithm library. Next, based on the access permissions, access the authorized equipment twin data stored in the twin data center (sensitive equipment data is authorized through the maintenance support digital twin platform to form data access permissions), and load it into the fault diagnosis algorithm and model. Next, perform the fault diagnosis process, including data preprocessing, feature extraction, and fault diagnosis steps. If the diagnosis result is a fault, perform fault isolation processing, update the virtual digital equipment, and optimize the fault diagnosis algorithm or model based on the feedback algorithm library of the fault isolation processing situation. If the diagnosis result is no fault, continue to perform online diagnosis based on real-time twin data.

## 6. Conclusions

In order to improve the efficiency of fault diagnosis and the interactive experience of the diagnosis process for clearly defined equipment routine faults, this paper proposes a digital twin-based equipment routine fault diagnosis model. On this basis, considering the good interactivity and user experience of IETM, a conventional equipment fault diagnosis scheme based on twin data and IETM was designed. This scheme utilizes the fault tree analysis method, taking real-time twin data of equipment as input, converting the fault tree into the fault DM of IETM, storing it in the IETM database to form a fault library, utilizing the interactivity of IETM, and combining the process DM with the fault DM for step-by-step fault diagnosis and isolation guidance operations, while providing maintenance operation guidance. In response to the situation where the fault symptoms are not completely consistent with the fault DM in IETM, a case analysis method is used to calculate the similarity between the fault symptom information provided by the real-time twin data of the equipment and the clearly defined fault symptom information in the fault library. The similarity threshold is set for judgment. If it is greater than the equal similarity threshold, it is considered that the fault represented by the real-time data of the equipment is consistent with the existing fault in the fault library. IETM pushes the corresponding fault DM for the corresponding fault diagnosis isolation guidance. Our further research focuses on the accuracy and speed of fault tree analysis and case similarity calculation, determining the threshold for case similarity, using a large prediction model to automatically generate fault tree analysis reasoning rules from equipment technical data, and combining it with a large language model for fault diagnosis applications [37]. Based on the fault tree and maintenance data of the equipment, generate fault DM and maintenance DM automatically through the large language models.

## Figures and Tables

**Table 1:** Comparison of fault diagnosis methods.

Methods	Principle	Accuracy	Advantage	Disadvantage	Scenarios
Physical model-driven approach	Accurate mathematical models, such as differential equations and state space models, are used to simulate system behavior.	75~89%.	Real-time performance is good, and the diagnostic results have clear physical significance, which can directly relate to the failure mechanism of specific components and provide a direct basis for maintenance decisions.	The operational mechanism of the equipment needs to be deeply understood, and precise modeling is difficult.	High safety fields such as aerospace engines and nuclear power control.
Knowledge-driven approach	The experience of domain experts is transformed into a computable rule system, which diagnoses equipment faults based on rules and monitoring data, including methods such as fault tree analysis (FTA), Fuzzy Inference Systems (FISs), and Bayesian networks (BNs).	The fault mode is known, and the diagnostic accuracy can reach 83% to 100%.	Low computational resource consumption, good real-time performance, clear knowledge of conventional faults, high diagnostic accuracy, and a simple modern causal diagram diagnostic reasoning process.	When the fault mode is unknown, the accuracy of fault diagnosis is low and the maintenance cost of the rule library is high.	Complex systems with unclear mechanisms or scarce data are particularly suitable for routine fault diagnosis of equipment with known failure modes.
Data-driven approach	Equipment fault diagnosis and prediction are carried out through feature extraction, training modeling, and testing of data, including signal processing and feature engineering, statistical learning and machine learning, and deep learning methods.	Simple components can reach 92–99%, while the accuracy of fault diagnosis for complex equipment is low.	The black box mode for fault diagnosis and prediction does not require knowledge of fault modes and physical mechanisms.	High consumption of computing resources, poor real-time performance, high dependence on data quality, poor interpretability of diagnostic results, and questionable confidence.	Equipment with rich and high-quality sensor data, unclear fault modes, and unclear physical mechanisms.
Hybrid intelligence method	Multiple diagnostic methods are integrated to break through the limitations of a single method, including signal model mixing, data knowledge collaboration, cross-domain feature fusion, optimization algorithm coupling, and other methods.	The accuracy of mixed methods in diagnosing complex equipment generally exceeds 90%.	The diagnostic accuracy is high, requiring a clear understanding of some fault modes and physical mechanisms, and the dependence on data quality is relatively low.	The consumption of computing resources is high, the real-time performance is poor, the interpretability of diagnostic results is higher than that of data-driven methods, and the confidence level is relatively high.	Complex equipment field.

**Table 2:** Correspondence of fault tree, XML, and IETM DM elements.

Fault Tree	XML Elements	IETM DM Elements
Top event nodes	<TopNode>	<dmRef>
Middle event nodes	<MidNode>	<dmNode>
Middle event nodes	<BotNode>	<dmNode>
And	@relation = and	<dmIf>
Or	@relation = or	<dmThenSeq>
Xor	@relation = xor	<dmElseSeq>
Non	@relation = not	<dmIf>

**Table 3:** Middle and basic events of gearbox malfunction.

Events	No.	Events	No.	Events	No.
Clutch slippage	M_1_	Gear bonding	M_16_	Abrasive wear	B_15_
Bomponent failure	M_2_	Poor material quality	B_1_	Poor assembly quality	B_16_
Oil leakage	M_3_	Excessive wear and tear	B_2_	Fatigue fracture	B_17_
Friction plate failure	M_4_	Poor lubrication	B_3_	Poor machining accuracy	B_18_
Oil hole blockage	M_5_	Oil quality issues	B_4_	The material becomes brittle	B_19_
Gear failure	M_6_	Insufficient oil pressure	B_5_	Severe overload	B_20_
Bearing failure	M_7_	Paper pad blockage	B_6_	Casting defects	B_21_
Axis malfunction	M_8_	Fatigue fracture	B_7_	Spring pad failure	B_22_
Box rupture	M_9_	Overload breakage	B_8_	Insufficient preload force	B_23_
Fastener malfunction	M_10_	Random breakage	B_9_	Mechanical vibration	B_24_
Seal aging	M_11_	Abrasive wear	B_10_	Seal aging	B_25_
The joint surface is not tight	M_12_	Low-speed wear	B_11_	Assembly damage	B_26_
The temperature is too high	M_13_	Corrosive wear	B_12_	Poor quality of oil seal	B_27_
Gear fracture	M_14_	Metal weld	B_13_	Poor processing quality	B_28_
Tooth surface wear	M_15_	High-temperature failure of lubricating oil	B_14_	Sealing failure	B_29_

**Table 4:** Probability of basic events in the aviation gearbox.

Events	B_1_	B_2_	B_3_	B_4_	B_5_	B_6_	B_7_	B_8_	B_9_	B_10_
Probability of occurrence	0.002	0.005	0.0014	0.001	0.005	0.008	0.0005	0.0014	0.0021	0.0018
Events	B_11_	B_12_	B_13_	B_14_	B_15_	B_16_	B_17_	B_18_	B_19_	B_20_
Probability of occurrence	0.0024	0.0018	0.0014	0.006	0.002	0.0004	0.0014	0.0006	0.0005	0.0018
Events	B_21_	B_22_	B_23_	B_24_	B_25_	B_26_	B_27_	B_28_	B_29_	
Probability of occurrence	0.0016	0.0006	0.0008	0.0006	0.0008	0.004	0.0004	0.0006	0.0006	

**Table 5:** Real-time twin data and technical requirements for the aviation gearbox at time t.

Parameter	Value	Requirement	Parameter	Value	Requirement
Friction pad material	Steel Q235	Steel Q235	Drive gear bearings	Normal	No damage
Friction plate deformation	Not have	No warping deformation	Oil debris	Not have	Impurities and paint shavings
Friction plate damage	Not have	surface damage	Oil circuit	Normal	Not blocked
Deposition on the surface of the friction plate	Not have	Salt, sediment, dirt	Remaining time of lubricating oil	5 months	0 ≤ t ≤ 6 months
Friction plate working temperature	100 °C	≤200 °C	Oil hole diameter	0.5 mm	0.4 ≤ d ≤ 0.6 mm
Friction pad moisture pollution	Not have	The gears are not rusted, and there is no oxidation, emulsification, or corrosion	Paper pad	Undeformed	Flat and undistorted
Friction plate vibration displacement	0.5 mm	0 ≤ l ≤ 0.8 mm	Oil hole	Smooth	Not blocked
Friction plate vibration velocity	1 mm/s	0 ≤ v ≤ 2 mm/s	Fatigue crack	0.01 mm	≤1.5 mm
Friction plate vibration acceleration	1 mm/s^2^	0 ≤ v ≤ 2 mm/s^2^	Concentrated stress	1200 MPa	≤1500 MPa
Friction plate noise	40 dB	≤80 dB	Load	600 KN/mm^2^	0 ≤ l ≤ 1500 MPa
Oil viscosity	15 w	10 w ≤ n ≤ 40 w	Random fracture of tooth tip	Not have	No random fracture at the tooth tip
Oil expiration date	2/5/2027	Current time ≤ expiration time	Random fracture of the tooth waist	Not have	No random fracture in the tooth waist
Lubricating oil grade	A4	≥A2	Random fracture of tooth root	Not have	No random fracture of tooth root
Oil pressure	0.3 MPa	0.12–0.3 MPa	Crack	Not have	Depth ≤ 0.03 mm
Oil level	12 mm	8 mm ≤ h ≤ 15 mm	Residual stress	100 KN/mm^2^	0 ≤ Rs ≤ 500 MPa
Oil pump blades	Normal	No damage	Gear tooth offset load	0.05 mm	0 ≤ OL ≤ 0.2 mm
Impact load	200 KN/mm^2^	0 ≤ IL ≤ 600 MPa	Oil viscosity	ISO VG46	≤ISO VG32
Diameter of foreign object	0.02 mm	d ≥ 0.05 mm	Backlash	0.04 mm	0 ≤ mc ≤ 0.03 mm
Number of abrasive particles	80 pieces/mL	≤50 pieces/mL	Tooth surface	Pit	Flat and smooth
Grinding particle diameter	0.002–0.015 mm	≤0.1 mm	Tooth surface wear	Not have	No wear and tear, no cracking

**Table 6:** Probability proportion of basic events.

Events	B_1_	B_2_	B_3_	B_4_	B_5_	B_6_
Probability proportion	0.03634183	0.09085458	0.02543928	0.01817092	0.09085458	0.14536733
Events	B_7_	B_8_	B_9_	B_10_	B_11_	B_12_
Probability proportion	0.00908546	0.02543928	0.03815892	0.03270765	0.0436102	0.03270765
Events	B_13_	B_14_	B_15_	B_16_	B_17_	B_18_
Probability proportion	0.02543928	0.10902549	0.03634183	0.00726837	0.02543928	0.01090255
Events	B_19_	B_20_	B_21_	B_22_	B_23_	B_24_
Probability proportion	0.00908546	0.03270765	0.02907347	0.01090255	0.01453673	0.01090255
Events	B_25_	B_26_	B_27_	B_28_	B_29_	
Probability proportion	0.01453673	0.07268366	0.00726837	0.01090255	0.01090255	

**Table 7:** Probability and probability proportion of intermediate events.

Events	M_1_	M_2_	M_3_	M_4_	M_5_	M_6_	M_7_	M_8_
Probability of malfunction occurrence	0.0224	0.0277	0.0064	0.0094	0.013	0.0174	0.0024	0.002
Probability proportion	0.407031	0.503337	0.116294	0.170807	0.236223	0.316176	0.04361	0.036342
Events	M_9_	M_10_	M_11_	M_12_	M_13_	M_14_	M_15_	M_16_
Probability of malfunction occurrence	0.0039	0.002	0.0052	0.0012	0.0012	0.0024	0.004	0.006
Probability proportion	0.070867	0.036342	0.094489	0.021805	0.021805	0.04361	0.072684	0.109026

**Table 8:** Gearbox fault tree and maintenance guide converted to IETM DM list.

DM	Type	Quoting DM	DM	Type	Quoting DM
T	process	M_1_, M_2_, M3, TM	TM maintenance	procedure	MM_1_,MM_2_,MM_3_
M_1_	fault	M_4_, M_5_, MM_1_	MM_1_ maintenance	procedure	MM_4_,MM_5_
M_2_	fault	M_6_, M_7_,M_8_, M_9_, M_10_, MM_2_	MM_2_ maintenance	procedure	MM_6_,MM_7_,MM_8_,MM_9_,MM_10_
M_3_	fault	M_11_, M_12_, MM_3_	MM_3_ maintenance	procedure	MM_11_,MM_12_
M_4_	fault	B_1_, B_2_, M_13_, MM_4_	MM_4_ maintenance	procedure	BM_1_,BM_2_,MM_13_
M_5_	fault	B_5_, B_6_, MM_5_	MM_5_ maintenance	procedure	BM_5_,BM_6_
M_6_	fault	M_14_, M_15_, M_16_, MM_6_	MM_6_ maintenance	procedure	MM_14_,MM_15_,MM_16_
M_7_	fault	B_15_, B_16_, MM_7_	MM_7_ maintenance	procedure	BM_15_,BM_16_
M_8_	fault	B_17_, B_18_, MM_8_	MM_8_ maintenance	procedure	BM_17_,BM_18_
M_9_	fault	B_19_, B_20_, B_21_, MM_9_	MM_9_ maintenance	procedure	BM_19_,BM_20_,BM_21_
M_10_	fault	B_22_, B_23_, B_24_, MM_10_	MM_10_ maintenance	procedure	BM_22_,BM_23_,BM_24_
M_11_	fault	B_25_, B_26_, B_27_, MM_11_	MM_11_ maintenance	procedure	BM_25_,BM_26_,BM_27_

**Table 9:** Gearbox fault tree and maintenance guide converted to IETM DM list—continued.

DM	Type	Quoting DM	DM	Type	Quoting DM
M_12_	fault	B_28_, B_29_, MM_12_	MM_12_ maintenance	procedure	BM_28_,BM_29_
M_13_	fault	B_3_, B_4_, MM_13_	MM_13_ maintenance	procedure	BM_3_,BM_4_
M_14_	fault	B_7_, B_8_, B_9_, MM_14_	MM_14_ maintenance	procedure	BM_7_,BM_8_,BM_9_
M_15_	fault	B_10_, B_11_, B_12_, MM_15_	MM_15_ maintenance	procedure	BM_10_,BM_11_,BM_12_
M_16_	fault	B_13_, B_14_, MM_16_	MM_16_ maintenance	procedure	BM_13_,BM_14_
B_1_	fault	BM_1_	BM_1_ maintenance	procedure	
B_2_	fault	BM_2_	BM_2_ maintenance	procedure	
B_3_	fault	BM_3_	BM_3_ maintenance	procedure	
B_4_	fault	BM_4_	BM_4_ maintenance	procedure	
B_5_	fault	BM_5_	BM_5_ maintenance	procedure	
B_6_	fault	BM_6_	BM_6_ maintenance	procedure	
B_7_	fault	BM_7_	BM_7_ maintenance	procedure	
B_8_	fault	BM_8_	BM_8_ maintenance	procedure	
B_9_	fault	BM_9_	BM_9_ maintenance	procedure	
B_10_	fault	BM_10_	BM_10_ maintenance	procedure	
B_11_	fault	BM_11_	BM_11_ maintenance	procedure	
B_12_	fault	BM_12_	BM_12_ maintenance	procedure	
B_13_	fault	BM_13_	BM_13_ maintenance	procedure	
B_14_	fault	BM_14_	BM_14_ maintenance	procedure	
B_15_	fault	BM_15_	BM_15_ maintenance	procedure	
B_16_	fault	BM_16_	BM_16_ maintenance	procedure	
B_17_	fault	BM_17_	BM_17_ maintenance	procedure	
B_18_	fault	BM_18_	BM_18_ maintenance	procedure	
B_19_	fault	BM_19_	BM_19_ maintenance	procedure	
B_20_	fault	BM_20_	BM_20_ maintenance	procedure	
B_21_	fault	BM_21_	BM_21_ maintenance	procedure	
B_22_	fault	BM_22_	BM_22_ maintenance	procedure	
B_23_	fault	BM_23_	BM_23_ maintenance	procedure	
B_24_	fault	BM_24_	BM_24_ maintenance	procedure	
B_25_	fault	BM_25_	BM_25_ maintenance	procedure	
B_26_	fault	BM_26_	BM_26_ maintenance	procedure	
B_27_	fault	BM_27_	BM_27_ maintenance	procedure	
B_28_	fault	BM_28_	BM_28_ maintenance	procedure	
B_29_	fault	BM_29_	BM_29_ maintenance	procedure	

**Table 10:** Fault diagnosis results.

Events	B_1_	B_2_	B_3_	B_4_	B_5_	B_6_	B_7_	B_8_	B_9_	B_10_	B_11_	B_12_	B_13_	B_14_	B_15_
State	0	0	1	0	0	0	0	0	0	1	0	0	0	0	0
Events	B_16_	B_17_	B_18_	B_19_	B_20_	B_21_	B_22_	B_23_	B_24_	B_25_	B_26_	B_27_	B_28_	B_29_	M_1_
State	0	0	0	0	0	0	0	0	0	0	0	0	0	0	1
Events	M_2_	M_3_	M_4_	M_5_	M_6_	M_7_	M_8_	M_9_	M_10_	M_11_	M_12_	M_13_	M_14_	M_15_	M_16_
State	1	0	1	0	1	0	0	0	0	0	0	1	0	1	0

## Data Availability

The data can be accessed from this manuscript.

## Abbreviations

The following abbreviations are used in this manuscript:

IETM	Interactive Electronic Technical Manual
DM	data model
FTA	fault tree analysis
KNN	K-Nearest Neighbors
PLC	Programmable Logic Controller
OPC UA	OPC Unified Architecture
FIM	Fault Isolation Manual
AMM	Aircraft Maintenance Manual
LRU	logic replaceable unit
SRU	shop replaceable unit
WebGL	Web Graphics Library
GAN	Generative Adversarial Network
SHAP	Shapley Additive exPlanations
LIME	Local Interpretable Model-agnostic Explanations
Grad-CAM	Gradient-weighted Class Activation Mapping
